# Outcomes of treatment for right atrial isomerism with functional single ventricle and extracardiac total anomalous pulmonary venous connection beyond neonatal period: Delayed surgical treatment, improving outcomes

**DOI:** 10.3389/fcvm.2022.914609

**Published:** 2022-12-20

**Authors:** Weidan Chen, Ye Lu, Li Ma, Shengchun Yang, Minghui Zou, Wenlei Li, Xinxin Chen

**Affiliations:** Cardiovascular Center, Guangzhou Women and Children's Medical Center, Guangzhou Medical University, Guangzhou, China

**Keywords:** right atrial isomerism, total anomalous pulmonary venous connection, heterotaxy syndrome, sutureless technique, single ventricle

## Abstract

**Objective:**

Total anomalous pulmonary venous connection (TAPVC) is frequently associated with right atrial isomerism (RAI), which is commonly complicated with an unbalanced atrioventricular canal with contralateral hypoplasia, complex systemic and pulmonary venous anatomy, and conotruncal abnormalities, resulting in increased risk of mortality. This study aimed to review the outcomes of delayed surgical treatment for patients with RAI complicated with functional single ventricle (FSV) and TAPVC at a single center.

**Methods:**

In this retrospective study, we reviewed the medical records of 24 consecutive patients with RAI complicated with FSV and TAPVC who underwent initial surgical palliation after 5-month old between September 2008 and June 2019. Demographic data, concomitant anomalies, age at initial palliation, and surgical interventions were extracted and analyzed using the Cox proportional hazard model to assess risk factors for mortality and the Kaplan-Meier method to assess survival.

**Results:**

The in-hospital mortality was 12.5% (three out of 24). The causes of death were pulmonary arterial hypertension and low cardiac output syndrome. Average follow-up was 65.2 ± 40.3 months (7–137 months). Another 4 patients died during the follow-up due to low cardiac output syndrome, protein-losing enteropathy and pulmonary arterial hypertension, respectively. Kaplan-Meier estimated survival at 1 and 5 years were 83.1 and 69.4%, respectively. Fontan completion was 45.8% (11/24). The mortality for patients with pulmonary venous obstruction (PVO) was 66.7% (4/6). Cox multivariate regression analysis indicated that preoperative PVO was the only risk factor for mortality (*p* = 0.032; hazard ratio, 10.000; CI 1.222–81.811).

**Conclusion:**

Outcomes of delayed surgical treatment for patients with RAI complicated with FSV and TAPVC have improved significantly. The survival and Fontan completion were higher. However, preoperative PVO was still the risk factor for mortality.

## Introduction

During the past 30 years, children with single ventricle lesions have demonstrated improved survival with the advent of early postnatal identification, innovative therapies, and staged surgical procedures culminating in the Fontan circulation (1–3). However, total anomalous pulmonary venous connection (TAPVC) is frequently associated with heterotaxy syndrome, especially right atrial isomerism (RAI), which commonly includes an unbalanced atrioventricular canal with contralateral hypoplasia, complex systemic and pulmonary venous anatomy, and conotruncal abnormalities, resulting in increased risk of mortality (4, 5).

RAI associated with TAPVC carries one of the worst outcomes in current surgical practice. Neonatal palliative surgery carries a high operative risk of early mortality. The report from the Society of Thoracic Surgeons Congenital Heart Surgery Database about patients with heterotaxy syndrome who underwent TAPVC repair at age of 90 days or younger confirmed the high mortality risk, particularly in patients with functionally univentricular physiology (4). Furthermore, surgical repair of RAI complicated with obstructed TAPVC during neonatal period is associated with frequent recurrence of pulmonary venous obstruction. Delayed surgical treatment may improve the outcomes. Delayed surgical treatment not only consists of inserting a stent to relief obstruction of TAPVC for selected critical neonates and perform the second stage operation after several months, but also means that surgical treatment occurs beyond the neonatal period for patients who are relatively stable. Even in developed countries, patients with TAPVC associated with a functional single ventricle exhibit high morbidity and mortality (6–11). The purpose of this study was to describe outcomes of delayed surgical treatment for patients with RAI complicated by TAPVC.

## Methods

The study protocol was reviewed and approved by the Institutional Review Board at Guangzhou Women and Children's Medical Center, and individual consent for the study was waived owing to its retrospective medical record review design. The databases of the Department of Cardiac Surgery were searched for patients with RAI and TAPVC who had undergone operative treatment. Medical records, operative notes, and all available electrocardiograms, echocardiography reports, and cardiac catheterization reports were reviewed. From September 2008 to June 2019, 24 consecutive patients with RAI, functional single ventricle and extracardiac TAPVC underwent initial surgical palliation after 5-month old at our center. Twenty-two patients (91.6%) were male and 2 (8.4%) were female. The median age at the time of the initial operation was 522 days (range 165–3,718 days), and median body weight was 8.45 kg (range 6–19.2 kg).

The type of TAPVC was supracardiac in 23 patients and infracardiac in one patient. Pulmonary venous obstruction (PVO) was found to coexist in six (6/24, 25%) patients. In 20 patients, there was a complete atrioventricular canal (CAVC), and in most cases, there was an unbalanced opening into the morphologic right ventricle. Atrioventricular valve regurgitation ≥ moderate was demonstrated by echocardiography in four patients. Sixteen patients had bilateral superior vena cava, usually without a connecting innominate vein. A typical double outlet right ventricle, with a muscular outlet conus under each great vessel associated with pulmonary atresia, was present in 4 patients. Patient characteristics are shown in Table 1.

**Table 1:** Characteristics of patients with right atrial isomerism and TAPVC.

Age, median [day, median (range)]	522 (165–3,718)
Male, *n*	22 (91.6%)
Weight [kg, median (range)]	8.45 (6–19.2)
TAPVC type, *n*	
Supracardiac	23
Infracardiac	1
CAVC	20
AVVR ≥ moderate	4
Pulmonary atresia	4
Pulmonary stenosis	17
PVO	6
Bilateral superior vena cava	16
Apicocaval juxtaposition	8

AVVR, atrioventricular valve regurgitation; CAVC, complete atrioventricular canal; PVO, pulmonary venous obstruction; TAPVC, total anomalous pulmonary venous connection.

### Surgical technique

TAPVC repair was performed in most patients during the initial palliation. Following median sternotomy, hypothermic cardiopulmonary bypass was performed. The ascending aorta was cannulated, and up to four venous cannulas were inserted into the superior vena cava, inferior vena cava (IVC), and hepatic veins when these did not drain into the IVC. Aortic cross clamping and cardioplegic arrest were performed. Repair of TAPVC was performed with the common pulmonary venous chamber and atrium anastomosis under mild hypothermia. A sutureless technique was used for most of the patients (including six patients with PVO). In patients who underwent a bidirectional Glenn procedure (BDG), the pulmonary artery was dissected and the anatomy of the systemic and pulmonary connections was inspected. Pulmonary arterioplasty was performed when necessary. The atrial septum was then opened in all patients. Modified Fontan (extracardiac Fontan) was likewise performed through a median sternotomy, with mild hypothermia and cardiopulmonary bypass. If there was no planned concomitant intracardiac procedure, the heart remained beating for the duration of the procedure. A total extracardiac Fontan was constructed using an 18–22 mm Gore-Tex tube graft anastomosed to the IVC and the ipsilateral pulmonary artery or the contralateral pulmonary artery. The route of Fontan connection was chosen according to our preference to obtain unobstructed flow of IVC and hepatic vein blood to the pulmonary artery without compromising pulmonary venous blood flow, or to obtain unobstructed flow of both vessels. A 3–4 mm fenestration was placed for all patients.

Data are presented as mean ± standard deviation or median and range, as appropriate. Cox proportional hazard models were used to analyze risk factors for survival. Estimated survival was determined by the Kaplan-Meier method based on the product-limit estimator, and 95% confidence intervals were constructed around curves using Greenwood's formula. Differences were considered statistically significant if the *P*-value was < 0.05.

## Results

One out of 24 patients underwent TAPVC repair and Blalock-Taussig (BT) shunt prior to BDG. Eighteen patients underwent TAPVC repair at the same time as BDG. Concomitant atrioventricular valvuloplasty was performed in three patients. However, two out of 18 suffered low cardiac output syndrome and hypoxemia, and underwent BDG takedown. TAPVC persisted in five patients who underwent BDG (*n* = 4) and one-stage modified Fontan (*n* = 1; Table 2). The mean cardiopulmonary bypass time was 154 ± 80 min; the mean aortic cross-clamping time (*n* = 20) was 54 ± 23 min.

**Table 2:** Procedure for patients with RAI and TAPVC.

**Procedure**	**Number**
**First stage**	
TAPVC repair + BT shunt	1
BDG	4
BDG + TAPVC repair	15^#^
BDG + TAPVC repair + AVR	3^§^
Modified Fontan	1
**Second stage**	
AV replacement	1
PVO repair + AVR	1
Modified Fontan	11*
**Third stage**	
Modified Fontan	1

AV, atrioventricular valve; AVR, atrioventricular valve repair; BDG, bidirectional Glenn; BT, Blalock-Taussig; PVO, pulmonary venous obstruction; TAPVC, total anomalous pulmonary venous connection.

^#^One patient suffered low cardiac output syndrome and hypoxemia, and underwent bidirectional Glenn takedown and underwent central shunt for low cardiac output syndrome and hypoxemia 1 week after initial palliation.

^§^One patient suffered low cardiac output syndrome and hypoxemia, and underwent bidirectional Glenn takedown.

^*^One was taken down after 2 days.

**Figure 1:**
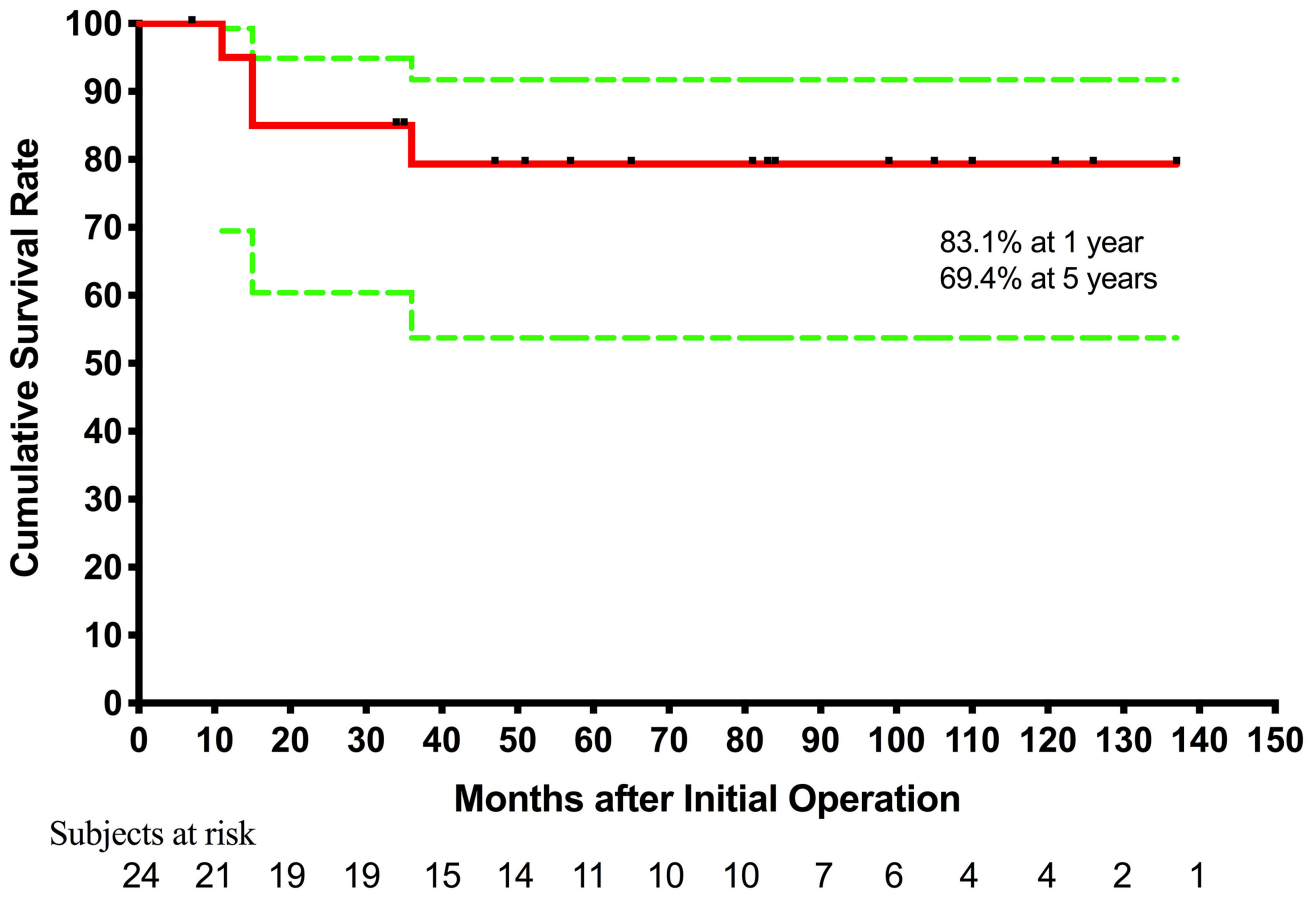
Cumulative incidence curve for estimated survival after initial palliation. Green lines denote 95% confidence intervals.

The hospital mortality rate was 12.5% (three out of 24). They all underwent bidirectional Glenn+ TAPVC repair at the first stage. One underwent atrioventricular valve repair simultaneously, and one (70.2-month old) underwent Glenn takedown because central venous pressure went up to 25 mmHg due to pulmonary arterial hypertension. The causes of death were pulmonary arterial hypertension and low cardiac output syndrome. In five patients, TAPVC wasn't repair for the sites of pulmonary veins returned near the atrium and seemed confluent. The mean follow-up period was 65.2 ± 40.3 months (7–137 months) and follow-up was complete in all surviving patients. Four patients died during the follow-up. One died of protein-losing enteropathy 11 months after initial operation. One patient underwent cutback of a stenotic orifice and atrioventricular valve replacement for recurrent PVO and severe atrioventricular valve regurgitation 11 months after initial palliation, and died of low cardiac output syndrome 4 months after the second operation. One (22.8-month old) underwent TAPVC repair and BDG takedown due to central venous pressure up to 25 mmHg, and underwent modified BT shunt 1 week later, but died of a nervous system complication after 15 months. Another one underwent modified Fontan but the Fontan conduit was taken down after 2 days, and died of low cardiac output syndrome due to intraoperative coronary artery injury. Of the 17 survivors, one underwent atrioventricular valve replacement for severe atrioventricular valve regurgitation 14 months after initial operation. Totally, 13 patients underwent modified Fontan operation and 11 survived. The age at modified Fontan operation was 55.7 ± 28.2 months (range from 33.6 to 137.4 months). Fontan completion was 45.8% (11/24), while two are awaiting Fontan procedure, and two with higher PVR and PAH diagnosed by catheterization are accepting targeted therapy for PAH. Kaplan-Meier estimated survival after initial palliation was 83.1 and 69.4% at 1 and 5 years, respectively (Figure 1).

Univariate analysis and multivariate analysis both indicated that PVO was a significant risk factor for mortality (*p* = 0.032; hazard ratio, 10.000; CI 1.222–81.811). Age, weight, CAVC, pulmonary atresia, apicocaval juxtaposition, and atrioventricular valve regurgitation were not significantly associated with mortality (Table 3).

**Table 3:** Analysis of risk factors for survival.

**Variable**	**Univariable**	**Cox multivariable analysis**		
	***P*-value**	**Hazard ratio**	***P*-value**	**95% CI**
Age	0.310			
Weight	0.320			
PA	0.160			
AJ	0.204			
PVO	0.020	10.000	0.032	1.222–81.811
CAVC	0.160			
AVVR	0.315			

AJ, apicocaval Juxtaposition; AVVR, atrioventricular valve regurgitation; CAVC, complete atrioventricular canal defect; PA, pulmonary atresia; PVO, pulmonary venous obstruction.

## Discussion

Despite improvements in outcomes of isolated TAPVC treatment (12–14), previous reports on the overall outcomes for children with a single ventricle and TAPVC have generally shown a poor prognosis (6–11). Mortality following surgical treatment of these patients has been reported to range from 20 to 52% (6–11, 15–17), although an improvement has been noted over time. Staged surgical palliation has become standard therapy in most centers. Previously identified risk factors for mortality have included mixed TAPVC, younger age at first operation, TAPVC repair before BDG, low birth weight, extracardiac anomalies or genetic syndromes, and extracorporeal membrane oxygenation or ventricular assist device support, among others (6–11). According to our recent experience with 24 patients with RAI with functional single ventricle and extracardiac TAPVC, in-hospital mortality following the initial palliation was only 12.5%, and survival after initial palliation was 83.1 and 69.4% at 1 and 5 years, respectively, over a mean follow-up of 65.2 months. The risk factor for death identified by multivariate analysis was PVO.

Of the many associated malformations found in patients with a single ventricle and TAPVC, those requiring the repair of extracardiac TAPVC at a younger age are considered important risk factors for mortality (7). This indicates coexisting obstruction of pulmonary venous drainage pathways. In this study, PVO was indicated to be a risk factor for mortality. To address this problem, a sutureless technique was used for most of the patients. Application of a sutureless technique for relief of postoperative PVO was expected to improve the outcomes (18). However, four of six patients with PVO died in this study, and recurrent PVO developed in another one patient. It is reported that stent implantation has been used for patients with preoperative pulmonary venous obstruction, and might be an alternative approach to avoid early TAPVC repair. However, there are fewer documented experiences related to this treatment approach and it has not been shown to improve overall outcomes (11). We also realize that whether the pulmonary veins are obstructive or not, some neonates or small infants suffer severe hypoxemia and metabolic acidosis, emergency TAPVC repair is enforced and the morbidity and mortality are higher.

As with other reports, most patients in this study (88%) exhibited right atrial isomerism (4, 5, 9). Right atrial isomerism has been recognized as one of the worst forms of congenital heart disease (CHD), with overall 5-year survival ranging from 30 to 74% (19–21). Even though this was not found to be the case in this study (*p* = 0.958), patients with right atrial isomerism are well-known to have a lifelong risk of overwhelming infection, common atrioventricular valve regurgitation, and arrhythmia, which can increase the mortality and morbidity of surgical treatment.

In the past three decades, great socio-economic development has taken place since the adoption of the reform and opening policy in China, and tremendous progress has been made in the field of medicine. However, shortages of basic equipment, facilities, and quality medical service at a grassroots level have led to misdiagnosis of CHD. Furthermore, poor awareness of public health issues results in delayed treatment, and most people from remote border districts are unable to afford the cost of treatment. As such, many patients undergo initial palliation at an older age. The median age at initial palliation in this study was 13.3 months, which is higher than other reports (median age from 4 to 69 days) (6–11). In most of the patients in our study, there was no severe PVO identified during the neonatal and early infant period, because coexisting obstruction of the pulmonary venous drainage pathways always requires early repair of extracardiac TAPVC. This likely reflects the stable nature of the patients who do not require earlier procedures. However, postoperative pulmonary arterial hypertension and increased pulmonary vascular resistance after initial palliation at an older age could increase mortality and lower the probability of Fontan completion. Only 11 (45.8%) patients achieved Fontan completion. In these patients, cyanosis disappeared and their general condition improved. The Fontan completion was only 26.3–46% for patients with functional single ventricle and extracardiac TAPVC who underwent initial operation at an early age (9–11, 22). For patients who did not complete the Fontan procedure, additionally close follow-up may be needed.

Given the dismal survival previously reported for patients with a functional single ventricle with TAPVC, some centers began to consider these patients for primary heart transplantation (HT) (23). Even though post-HT survival has improved for patients who failed to achieve Fontan completion, it is obviously limited by a shortage of donor organs and involves lifelong immunosuppressive therapy with its attendant risks and expenses (24, 25). The feasibility of transplantation for children is still uncertain in China.

## Limitations

Two major limitations of the present study are its retrospective nature and that the relatively small number of patients included in the study makes it difficult to identify risk factors for mortality since the statistical power to discern differences is relatively low. Although all patients had clear documentation of the preoperative and postoperative variables, other interesting variables such as pulmonary vascular resistance and pulmonary arterial pressure were less well-documented and could not be examined in relation to the outcomes. Furthermore, there wasn't a comparative group with patients who underwent repair as neonates or before 5 months of age.

## Conclusion

Surgical treatment can now be performed in patients with RAI with functional single ventricle associated and extracardiac TAPVC, with improving results. Initial palliation at an older age can be expected to lead to higher survival and higher probability of Fontan completion. Prompt and aggressive treatment may improve the outcomes for patients who underwent initial palliation at an older age.

## Data availability statement

The original contributions presented in the study are included in the article/supplementary material, further inquiries can be directed to the corresponding author.

## Ethics statement

The studies involving human participants were reviewed and approved by Guangzhou Women and Children's Medical Center. Written informed consent to participate in this study was provided by the participants' legal guardian/next of kin.

## Author contributions

All authors listed have made a substantial, direct, and intellectual contribution to the work and approved it for publication.
